# TAAR1 dependent and independent actions of the potential antipsychotic and dual TAAR1/5-HT_1A_ receptor agonist SEP-363856

**DOI:** 10.1038/s41386-022-01421-2

**Published:** 2022-09-13

**Authors:** Marcus Saarinen, Ioannis Mantas, Ivana Flais, Richard Ågren, Kristoffer Sahlholm, Mark J. Millan, Per Svenningsson

**Affiliations:** 1 Department of Clinical Neuroscience, Karolinska Institutet, Stockholm, Sweden; 2 Basal and Clinical Neuroscience, King’s College London, London, UK; 3 Department of Neuroscience, Karolinska Institutet, Stockholm, Sweden; 4 Department of Integrative Medical Biology, Wallenberg Centre for Molecular Medicine, Umeå University, Umeå, Sweden; 5 Neuroinflammation Therapeutic Area, Institut de Recherches Servier, Centre de Recherches de Croissy, Paris, France and Institute of Neuroscience and Psychology, College of Medicine, Vet and Life Sciences, Glasgow University, Scotland, Glasgow, UK

**Keywords:** Cellular neuroscience, Schizophrenia

## Abstract

SEP-363856 (SEP-856) is a novel antipsychotic under clinical development. It displays a unique pattern of receptor interaction, with only weak (partial agonist) activity at dopamine D_2_ receptors, yet more potent agonist activity at the trace amine associated receptor (TAAR1) and 5-hydroxytryptamine 1 A receptor (5-HT_1A_). Nonetheless, these observations await independent confirmation and more detailed characterization of the in vitro and in vivo actions of SEP-856 at TAAR1 and 5-HT_1A_ receptors would be instructive. Herein, we employed luminescence complementation technology in heterologous live cell systems, confocal microscopy, voltage clamp electrophysiology, behavioral readouts and TAAR1 knockout (KO) mice to study SEP-856 in further detail. We provide evidence for the ability of SEP-856 to activate TAAR1 at the surface plasma membrane, and show that this interaction results in Gα_s_ recruitment (pEC_50_: 6.08 ± 0.22 E_MAX_: 96.41% ± 15.26) and by extension, to G-protein inwardly rectifying potassium (GIRK) channel activation. Using TAAR1-KO mice, we find TAAR1 to be indispensable for SEP-856 control of body temperature, baseline locomotion reduction and for “antipsychotic-like” efficacy as characterized by a reversal of dizocilipine (MK-801) mediated disruption of pre-pulse inhibition. Conversely, the inhibition by SEP-856 of MK-801 induced locomotion was unaffected in TAAR1 KO mice. SEP-856 behaved as a low-potency, partial agonist at the 5-HT_1A_ receptor, while it partially inhibited recruitment of D_2_ receptor-coupled Gα and GIRK by DA and acted as a weak partial agonist with low potency at the same receptor when applied alone. Our findings corroborate and extend previous observations on the molecular substrates engaged by this unique, dual TAAR1/5-HT_1A_ receptor agonist and potential antipsychotic that could prove to have major advantages in the treatment of schizophrenia and other psychotic disorders.

## Introduction

Schizophrenia and other psychotic disorders affect over 20 million people worldwide [1]. Antipsychotic medications are the mainstay of pharmacological treatment for these debilitating mental afflictions. Pioneering work in the 1970 s identified the dopamine D_2_ receptor as a primary target of all first generation antipsychotics and was a pivotal advancement in neuropharmacology, even giving the D_2_ receptor the temporary name”antipsychotic receptor” [2–4]. Up until today, all FDA-approved antipsychotics act to some degree via the dopamine D_2_ receptor. Unfortunately, centrally-acting dopamine D_2_ receptor blockade can have serious side effects [5]. For example, extrapyramidal symptoms and hyperprolactinemia are common side effects of first-generation antipsychotics (“neuroleptics”) acting as potent D_2_ receptor antagonists [5, 6]. Clozapine and “atypical” antipsychotics like olanzapine and quetiapine target a broader range of receptors, in particular 5-HT_2A_ receptors which they more potently occupy than D_2_ receptors [7]. Some of these agents like aripiprazole and cariprazine behave as partial agonists at D_2_ receptors [8, 9]. The clinical effectiveness of these agents is however, still limited, in particular against negative and cognitive symptoms. Accordingly, there is substantial interest in *mechanistically novel* and therapeutically more effective classes of antipsychotic agents.

In this light, it is of considerable importance that, in 2019, Sunovion together with PsychoGenics published data on a behavioral phenotypic screen designed to detect non-dopaminergic antipsychotics [10]. The compound named SEP-856 was proposed to act through 5-HT_1A_ receptors and most interestingly, TAAR1. SEP-856 has subsequently been named Ulotaront and is being developed (Phase 1 3) by Sunovion who reported it to be clinically effective in the treatment of schizophrenia [11–13]. Additionally, a TAAR1 partial agonist (Ralmitaront), developed by Hoffmann-La Roche is currently under investigation in a Phase II clinical trial for the treatment of schizophrenia (ClinicalTrials.gov ID: NCT03669640) with another separate Phase II trial prematurely terminated (ID: NCT04512066).

TAAR1 is a member of the G-protein coupled receptor (GPCR) superfamily, belonging to the class A (rhodopsin like) receptors [14]. As its name suggests, various aminergic compounds such as trace amines and several classes of psychoactive agents activate TAAR1 [15]. While occurring in several peripheral organs, TAAR1 is highly expressed in the brain [16, 17]. It is enriched in monoaminergic nuclei, such as the ventral tegmental area (VTA), substantia nigra pars compacta (SNc), dorsal raphe nucleus, and the nucleus of the solitary tract. It is also present in the prefrontal cortex, entorhinal cortex, hypothalamus, and amygdala [18, 19]. TAAR1-KO mice display an increase in the firing rate of VTA and dorsal raphe neurons, a phenomenon which can be replicated in wildtype (WT) mice via local application of the TAAR1 antagonist EPPTB [20]. This suggests that TAAR1 exerts a tonic, inhibitory effect on the activity of dopaminergic and serotonergic neurons. Furthermore, TAAR1-KO mice display behavioral hypersensitivity and more pronounced increases in extracellular levels of monoamines in projection regions such as the striatum upon exposure to psychostimulants [21, 22]. Conversely, agonist stimulation of TAAR1 blunts the actions of psychostimulants [23]. Collectively, these observations support the potential utility of TAAR1 agonists as antipsychotics.

The 5-HT_1A_ receptor, also a Class A GPCR, is a brain-enriched member of the serotonergic receptor family [14, 17]. 5-HT_1A_ receptors are expressed on both serotonin neurons and non-serotonin neurons. In serotonergic neurons, the 5-HT_1A_ receptor exerts an autoinhibitory effect, decreasing cell firing and 5-HT release [24, 25]. In addition, 5-HT_1A_ receptors are highly expressed in several post-synaptic sites such as the cortex and hippocampal formation [26] where it regulates mood [27] and cognitive processes [28]. As mentioned above, many atypical antipsychotics antagonize 5-HT_2A_ receptors, yet there is ample evidence that partial agonist actions at 5-HT_1A_ receptors contribute to the functional profiles of many approved antipsychotics like aripiprazole, cariprazine, brexpiprazole, ziprasidone, and possibly lurasidone [29]. Taken together, partial 5-HT_1A_ receptor agonism, at least when combined with antagonist or partial agonists properties at D_2_ receptors, is considered a favourable feature for the treatment of schizophrenia [8, 30].

The purpose of this work was to further characterize the interaction of SEP-856 with TAAR1 and 5-HT_1A_ receptors and to further understand how this molecular signature influences its potential antipsychotic properties in vivo. In particular, we utilized a novel, codon-optimized TAAR1 in Expi293F cells to efficiently study the G-protein coupling profile of this receptor along with another unexplored signaling pathway of SEP-856, TAAR1 mediated GIRK channel activation, using oocyte electrophysiology. We then used TAAR1 KO mice to study the in vivo actions of SEP-856 with various clinically relevant, behavioral models of antipsychotic-like activity. The data presented here provide compelling evidence that mainly TAAR1, and possibly 5-HT_1A_ receptors are involved in the action of SEP 856, underpinning a potentially novel mechanism of antipsychotic activity.

## Materials and methods

HEK293T and Expi293F cells were maintained according to manufacturer’s conditions. G-protein recruitment was measured using a described system [31] in Expi293F cells. Oocytes were prepared and electrophysiological recordings done as previously described [32]. All animal experiments were approved by the Karolinska Institutet Animal Care and Use Committee according to Swedish guidelines in full compliance with European requirements. WT and TAAR1-KO [21, 22] mice were housed and behavioral experiments were performed as described in the Supplementary Methods. More information on all experimental methods included in this study including drug preparation, molecular biology, transfections, confocal imaging, flow cytometry, signaling assays and data analysis are available in detail in the Supplementary Methods.

## Results

### In vitro work

TAAR1 pharmacology has been traditionally difficult due to very poor receptor expression and cell surface availability, even in heterologous overexpression systems. Furthermore, in vivo TAAR1 expression levels are low as determined by many online sequencing databases. To overcome this obstacle in order to elucidate the functions of SEP-856 on TAAR1, we generated two mammalian codon optimized constructs. One contained the first nine N-terminal amino acids of the β2-adrenergic receptor (herein referred to as β-TA1), a strategy originally published by the Gainetdinov lab to increase cell surface expression [33], and one without (WT-TA1). Each construct contained triple hemagglutinin (HA) epitopes on the N-terminus with flexible linkers and C-terminal SmBiT tag (Supplementary Fig. 1). Receptor activation was assayed using the complementary luminescence (NanoBiT [31]) system between SmBiT-tagged TAAR1 and mini-G proteins fused to LgBiT, whereby receptor activation induces G-protein recruitment and thus reconstitutes a functional NanoLuc enzyme, yielding luminescence in the presence of the NanoLuc substrate luciferin.

**Fig. 1:**
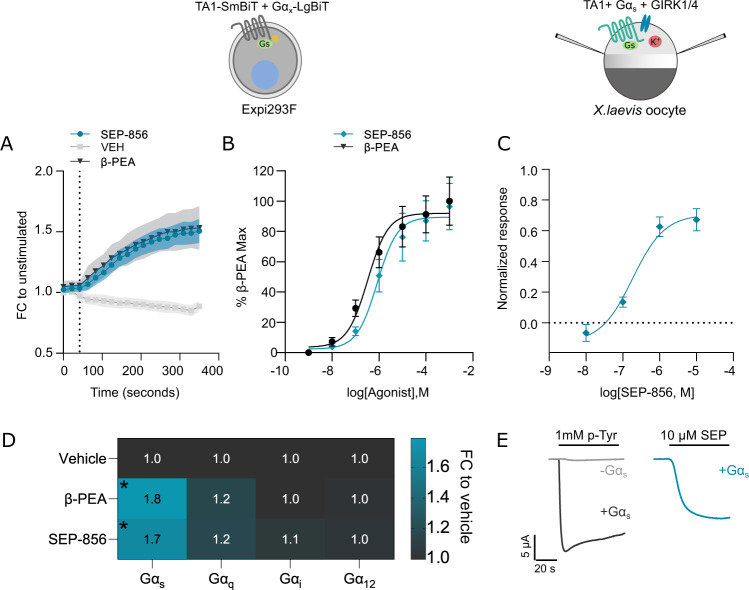
SEP-856 induced TAAR1 G-protein recruitment. All quantifications shown are generated using three independent samples consisting of duplicates. **A** Representative time series of WT-TA1- Gα_s_ recruitment after the addition of 1 mM SEP-856, β-PEA or VEH with the dotted line indicating time of compound addition. Shown as fold change (FC) of the average luminescence signal relative to VEH control (before addition of compounds). Outlines indicate error bars (S.E.M). **B** Concentration-response curves for both β-PEA- and SEP-856-induced Gα_s_ recruitment at WT-TA1. The pEC_50_ values for β-PEA and SEP-856 are 6.49 ± 0.23 and 6.08 ± 0.22, respectively. Error bars indicate S.E.M. **C** Concentration-response curve of SEP-856 in TAAR1- and Gα_s_ expressing oocytes normalized to the maximal response elicited by 1 mM p-tyramine (pEC_50_: 6.74 ± 0.43 E_MAX_: 67.15% ± 21.59). **D** Heat plot of the fold change for β-PEA- and SEP-856-induced Gα recruitment at WT-TA1. Compound-treated samples were compared to VEH controls using a two-way ANOVA which showed a significant interaction between G-protein recruitment and treatment (G-protein recruitment×treatment: F(6, 24) = 10.34, *p *< 0.0001 *p < 0.05, VEH vs Treated, Dunnett’s post hoc test.  **E** Representative traces of p-tyramine-induced GIRK currents in TAAR1-expressing oocytes in the presence (dark grey) and absence (light grey) of Gα_s_. Representative trace of GIRK currents evoked by 10 µM SEP-856 in an oocyte expressing TAAR1 and Gα_s_ (teal).

To examine the pharmacology of SEP-856, we began with interrogation of its effects on TAAR1. For all signaling experiments, we used the Expi293F cell line, which is grown in serum free media and thus avoids desensitization of the receptor via potential trace amines or serotonin present in animal serum. We first tested the ability of SEP-856 and the most potent suggested endogenous agonist, β-Phenethylamine (β-PEA), to recruit mini-G proteins through TAAR1 activation. Both compounds induced significant increases in Gα_s_ recruitment to WT-TA1 (Fig. 1A, Supplementary Fig. 2) and concentration-response curves for both compounds revealed similar agonist profiles (Fig. 1B). Additionally, no recruitment was observed with Gα_q_, Gα_i_ or Gα_12_ (Fig. 1C). We attempted to measure β-Arrestin recruitment at TAAR1, however neither of our β-Arrestin constructs (with C or N terminal LgBiT tags) indicated a successful agonist response when paired with the WT-TA1 construct (Supplementary Fig. 3B). Next, we tested several reported ligands of varying profiles to better rank agonists (Supplementary Fig. 4) to compare SEP-856 with, via Gα_s_ coupling. β-PEA, p-Tyramine and 3-methoxytyramine (3-MT) all acted as full agonists whereas dopamine was a partial agonist and norepinephrine acted as a very weak partial agonist. Moreover, β-TA1 yielded an increased luminescent signal and response to norepinephrine (Supplementary Fig. 4D). These experiments indicate that SEP-856 acts as a potent full agonist on TAAR1, acting specifically via Gα_s_ recruitment (pEC_50_: 6.08 ± 0.22 E_MAX_: 96.41% ± 15.26). In agreement with Dedic et al. [10], we find SEP-856 to be of slightly less potency than β-PEA (pEC_50_: 6.49 ± 0.23) on TAAR1, but of greater potency than p-Tyramine (pEC_50_: 5.65 ± 0.06, Supplementary Fig. 4E, F). Finally, to investigate an alternative aspect of TAAR1 downstream signaling, we expressed the codon optimized human TAAR1 construct in *Xenopus laevis* oocytes This permitted assessment of the ability of SEP-856 to induce GIRK channel activation [20]. The reference agonist p-tyramine elicited GIRK current responses only in oocytes expressing exogenous Gα_s_ (Fig. 1E). In the same oocytes, SEP-856 acted as a partial agonist (pEC_50_: 6.74 ± 0.43 E_MAX_: 67.15%  ± 21.59 relative to p-tyramine) of human TAAR1 (Fig. 1D).

**Fig. 2:**
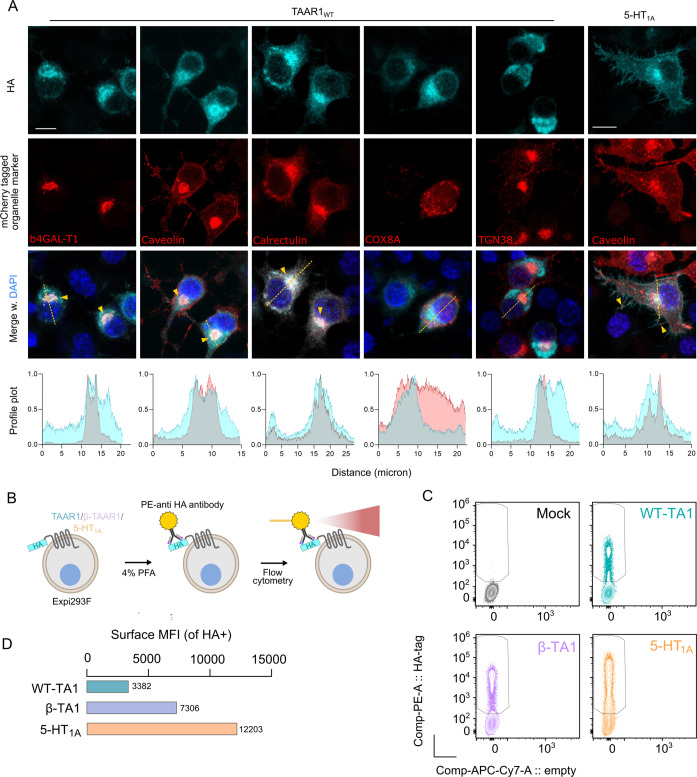
Subcellular localization of WT-TA1. **A** Representative images captured using a laser scanning confocal microscope of HA-stained TAAR1 (first row) together with various subcellular localization markers tagged with the constitutively fluorescent protein mCherry (second row). Right: example of HA-stained 5-HT_1A_ receptor together with the Golgi-surface plasma membrane protein Caveolin. Merged images of HA-stained receptor and localization marker are in the third row together with the nuclear marker DAPI. At least three different groups of cells were imaged for each condition. Yellow arrows indicate areas of notable co-localization between HA-stained receptor and the respective localization marker. Dashed lines indicate the cross section that was used to generate the profile plots shown below which graph the normalized intensity of the channel (Y axis, cyan: WT-TA1 and red: organelle marker) across the section (X axis). Cell surface receptor levels were measured using flow cytometry (**B**) with the staining intensity of surface labeled HA-tagged receptor shown in **C**. **D** depicts the mean fluorescence intensity of the surface stained receptors. Three separate batches of transfected cells for each receptor construct were combined, stained and analyzed as one.

After establishing that SEP-856 acts as a potent agonist on TAAR1 via Gα_s_ and having functional wildtype TAAR1 receptor expression, we sought to identify whether the construct is able to signal from the surface plasma membrane. This was important, as cell compartment specific signaling can give rise to various different outcomes as opposed to “standard” cell surface receptor signaling [34], which has been recently demonstrated for TAAR1 [35]. Likewise, the localization of TAAR1 has been difficult to pinpoint, with studies indicating intracellular, endoplasmic reticulum (ER) [33, 35], and more recently, ciliary localization in a thyroid cell line [36]. We reasoned that (1) since we observed rapid receptor activation by cell membrane- impermeable compounds such as dopamine and norepinephrine (2) TAAR1 is capable of GIRK channel activation, we should be able to detect TAAR1 at the cell surface plasma membrane. To address this issue, we first turned to confocal microscopy and expressed the WT-TA1 construct in HEK293T cells together with various cell organelle markers carrying a fluorescent mCherry tag (Fig. 2A). Antibody staining for the N terminal HA epitope tags on WT-TA1 revealed that the receptor is mainly retained within the cell, consistent with earlier reports. We observed co-localization with several organelle markers, most evident in the endoplasmic reticulum (ER) and the Golgi apparatus (Fig. 2A). No co-localization was observed with the mitochondrial marker COX8A, and co-localization with the surface plasma membrane/Golgi network protein Caveolin was mostly seen in the Golgi. Surprisingly, poor co-localization was observed with the trans-Golgi network marker TGN38. In comparison with the 5HT_1A_ receptor that displayed obvious cell surface expression, we failed to observe strong WT-TA1 signal of the surface plasma membrane. We reasoned that flow cytometry would provide a more sensitive and quantitative system to test TAAR1 surface expression. We used both TAAR1 constructs, since the β-TA1 construct should serve as a good positive control of TAAR1 cell surface expression, along with the 5-HT_1A_ receptor to compare the TAAR1 constructs against a more “typical” GPCR with a signal peptide for enhanced expression. Expi293F cells overexpressing these constructs or an empty pcDNA vector were fixed with formaldehyde and stained with PE-conjugated anti-HA antibodies without permeabilizing agents to exclusively label only cell surface available TAAR1 (Fig. 2B, Supplementary Fig. 9). We observed a clear presence of anti-HA labeled signal in WT-TA1 which was not present in mock (pcDNA) control cells (Fig. 2C). Furthermore, measures of the mean fluorescence intensity (an indicator of relative receptor surface density) of each labeled cell showed that as expected, the β-TA1 construct roughly doubled the staining intensity, whereas the signal from 5-HT_1A_ receptor expressing cells was considerably higher than either TAAR1 construct (Fig. 2D).

**Fig. 3:**
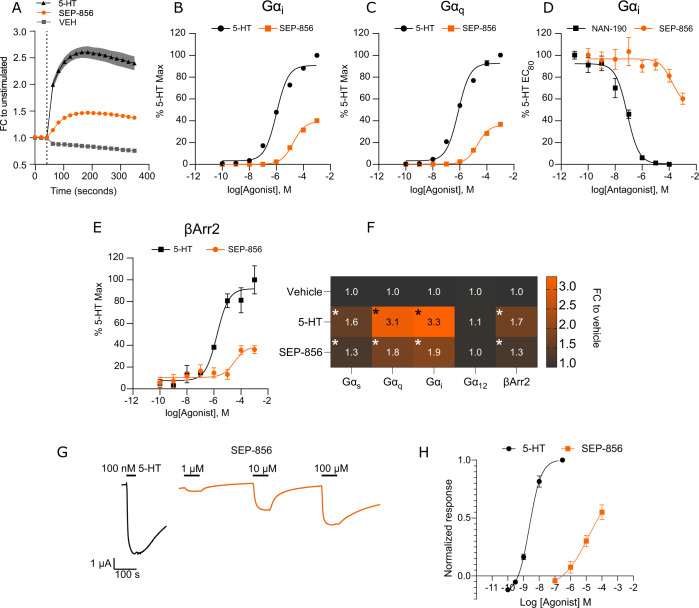
In vitro signaling profile of 5-HT1A by SEP-856. **A** Time course of Gα_i_ recruitment to 5-HT_1A_ in response to SEP-856 or 5-HT (1 mM, *n* = 3). Concentration-response curves for G-protein coupling to 5-HT_1A_ are shown in (**B**, pEC_50_ 5-HT:5.99 ± 0.07) and (**C**, pEC_50_ 5-HT:6.13 ± 0.07), (*n* = 3). **D** Concentration-response curve of SEP-856 or NAN-190 (pIC_50_: 7.1 ± 0.12) for inhibition of Gα_i_ coupling induced by co-administration of 5-HT (*n* = 3). **E** Concentration-response curves for β-Arrestin2 recruitment induced by 5-HT (pEC_50_: 5.78) and SEP-856 (*n* = 3). **F** Heat plot for maximal ligand-induced Gα and β-Arrestin2 recruitment shown as a fold change as compared to VEH-treated samples (*n* = 3, two-way ANOVA, G-protein / β-Arrestin2 recruitment×treatment: F_(8, 30)_=146.8, *p* < 0.0001) *p < 0.05, VEH vs Treated, Dunnett’s post hoc test). **G** Representative traces of 5-HT_1A-_evoked GIRK currents upon administration of 100 nM 5-HT compared to increasing doses of SEP-856. **H** Concentration-response curves for 5-HT_1A_-evoked GIRK currents by administration of 5-HT (*n* = 7, pEC_50_: 8.63 ± 0.05). SEP-856 (*n* = 5). Responses to SEP-856 were normalized to the response evoked by 100 nM 5-HT. All error bars indicate S.E.M.

Next, we sought to expand on the pharmacology between SEP-856 and the 5-HT_1A_ receptor since both SEP-856 binding and agonism was reportedly the greatest for the 5-HT_1A_ receptor after TAAR1 in a screen involving several aminergic receptors [10]. Unlike at TAAR1, SEP-856 proved to be a low-potency 5HT_1A_ receptor partial agonist when compared with the endogenous agonist serotonin (Fig. 3A). Like serotonin, SEP-856 was able to induce recruitment of Gα_q_ (E_MAX_: 36.67%  ± 1.79), Gα_i_ (E_MAX_: 40.11%  ±  0.70), and to a lesser extent, Gα_s_ (E_MAX_: 47.47%  ±  4.98) (Fig. 3B and C, Supplementary Fig. 5). A similar promiscuous coupling profile of the 5-HT_1A_ receptor is supported by several studies [37, 38]. However, the affinity for recruitment at all three G-protein subtypes was several orders of magnitude lower than that of serotonin. The potential for serotonin antagonism due to the partial agonist profile of SEP-856 on the 5-HT_1A_ receptor was also evaluated using an EC_80_ concentration of serotonin in this assay co-administered with varying concentrations of SEP-856 (Fig. 3D). SEP-856 was able to inhibit serotonin induced Gα_i_ coupling to a modest extent (39.75% ± 8.93) at the highest concentration tested. We then performed β-arrestin recruitment assays to investigate any further potential signaling bias of SEP-856 (Fig. 3E). β-arrestin recruitment by SEP-856 revealed a similar partial agonist profile (E_MAX_:36.05% ± 6.30). To further validate the low-potency partial agonist profile of SEP-856 on the 5-HT_1A_ receptor, we measured Gβγ mediated GIRK activation using *Xenopus laevis* oocytes since 5-HT_1A_ activation is known to robustly activate GIRK channels. Also in this assay system, SEP-856 was of lower potency and efficacy (E_MAX_: 55.03% ± 14.09) than serotonin (pEC_50_:8.62 ± 0.05, Fig. 3G, H), indicating that SEP-856 is able to induce GIRK channel mediated currents through the 5-HT_1A_ receptor at high concentrations, presumably due to Gα_i/o_ coupling.

**Fig. 4:**
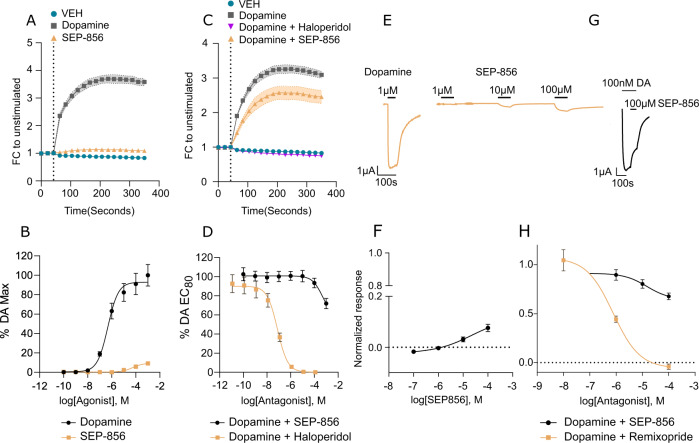
SEP-856 lacks significant activity at the D2 receptor. **A** Time series of Gα_i_ recruitment to the D_2_ in response to SEP-856 or dopamine in the nanoluciferase complementation assay (1 mM, *n* = 3). **B** Concentration-response curves for SEP-856 or dopamine at the D_2_ receptor in the nanoluciferase complementation assay (*n* = 3 pEC_50_ Dopamine: 6.33 ± 0.09). **C** Time series of dopamine mediated Gα_i_ recruitment inhibition to the D_2_ by haloperidol or SEP-856. **D** Concentration-response curves for haloperidol and SEP-856 co-applied with 2 µM dopamine in the nanoluciferase complementation assay (*n* = 3, pIC_50_: 7.1 ± 0.07 for haloperidol). **E** Representative traces of dopamine- and SEP-856-induced GIRK currents in D_2_ expressing oocytes. **F** Concentration-response curve SEP-856 in D_2_–expressing oocytes normalized to the maximal response elicited by 1 µM dopamine (*n* = 4, E_MAX_: 7.61% ± 1.69, measured as maximum response relative to 1 µM dopamine). **G** Representative traces of SEP-856 and 100 nM dopamine co-administration. **H** Dose response of either Remoxipride or SEP-856 together with dopamine, normalized to the response to 100 nM DA (Hill slope = −1, *n* = 3–6). All error bars indicate S.E.M.

Finally, to examine any influence of SEP-856 on dopamine D_2_ receptor activity, we began by using the G-protein recruitment assay to measure the degree of Gα_i_ interaction with the D_2_ receptor after stimulation with SEP-856 compared with the endogenous agonist dopamine. The results revealed that SEP-856 stimulation resulted in very limited Gα_i_ recruitment as opposed to dopamine (Fig. 4A). The concentration-response of SEP-856 implies it acts as a low potency partial agonist (E_MAX_: 9.14 ± 0.86, Fig. 4B) on the dopamine D_2_ receptor. Next, we tested SEP-856’s potential as an antagonist. SEP-856 was able to partially reduce the degree of Gα_i_ recruitment when co-administered with an EC_80_ of dopamine compared to dopamine alone (Fig. 4C). The classical typical antipsychotic haloperidol, on the other hand, was able to fully block and prevent any Gα_i_ response to dopamine stimulation. A dose-response of either drugs administered with dopamine revealed SEP-856 works as a weak antagonist (~30% inhibition of DA EC_80_ with 1 mM) on the D_2_ receptor (Fig. 4D). As D_2_ receptor activation can also evoke robust GIRK channel activation, we applied SEP-856 to *Xenopus laevis* oocytes expressing the D_2_ receptor and GIRK1/4 channel subunits. GIRK current responses to SEP-856 application were normalized to the current evoked by 1 µM dopamine, which is known to elicit a maximal response under the assay conditions used [32]. SEP-856 elicited limited GIRK activation only at high micromolar concentrations (Fig. 4E, F). As other D_2_ partial agonists, such as aripiprazole have been reported to antagonize dopamine-evoked GIRK activation [39], we sought to investigate the antagonist profile of SEP-856 in GIRK activation. At 100 µM, SEP-856 partially (32.24% ± 7.46%) blocked dopamine-induced GIRK activation. In comparison, remoxipride, a known low-affinity D_2_ antagonist [40], fully inhibited the GIRK response to dopamine (Fig. 4G, H).

### In vivo work

**Fig. 5:**
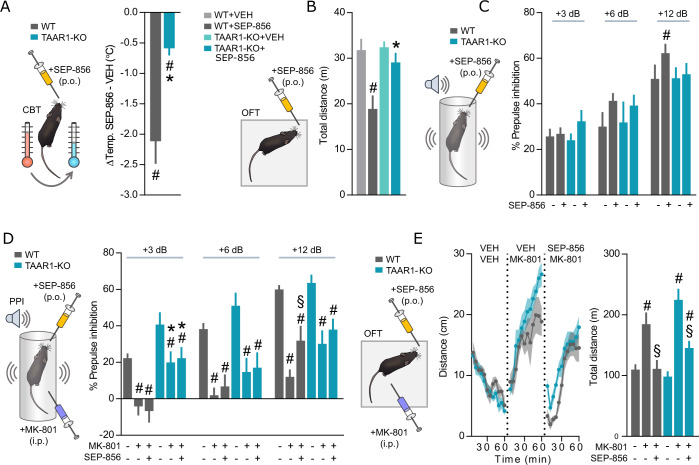
In vivo evaluation of SEP-856 antipsychotic potential in WT and TAAR1-KO mice. **A** CBT change was evaluated in both genotypes WT (*n* = 21), TAAR1**-**KO (*n* = 17) following oral SEP-856 (10 mg/kg) administration (*p = 0.0002, WT vs KO Mann Whitney test, ^#^*p* = 0.0001, One sample t-test with mean set to 0). **B** First 10 min of OFT in both genotypes after the oral administration of SEP-856 (10 mg/kg) (WT *n* = 5-6, TAAR1-KO *n* = 4-5, #*p* = 0.009, WT-Sal vs WT-SEP-856, *p = 0.047, WT-SEP-856 vs TAAR1-KO-SEP-856, 2-way ANOVA, Sidak’s post hoc-test. **C** Effects of SEP-856 on PPI in both genotypes WT (*n* = 21), TAAR1-KO (*n* = 16) compared against VEH only (^#^*p* = 0.0045, VEH vs SEP-856, Sidak’s post-hoc). **D** SEP-856 reversal of MK-801 PPI disruption, *n* = 10-13 mice for all groups (*p < 0.01, WT vs TAAR1-KO; ^#^*p* < 0.05, VEH-VEH vs VEH -MK-801; ^§^*p* < 0.05, VEH-MK-801 vs SEP-856-MK-801, Sidak’s post-hoc). **E** SEP-856 pre-treatment on MK-801 (0.4 mg/kg) induced hyperlocomotion was determined in the open field test in WT (*n* = 7) and TAAR1-KO (*n* = 7) mice (^#^*p* < 0.01, VEH- VEH vs VEH -MK-801; ^§^*p* < 0.01, VEH -MK-801 vs SEP-856-MK-801, Sidak’s post-hoc). Time bins shown are in 5-minute intervals.

After examining SEP-856 effects in vitro we decided to probe its effects in vivo using WT and TAAR1-KO mice to pinpoint which behavioral parameters are TAAR1 dependent. It has been reported that selective TAAR1 agonists decrease core body temperature (CBT) [41]. Thus, to validate that SEP-856 exerts a functional physiological effect in vivo through TAAR1, we first tested the ability of SEP-856 to decrease CBT. Accordingly, SEP-856 (10 mg/kg) was able to reduce the CBT of WT mice by 2 ^◦^C, while TAAR1-KO mice responded with a much smaller, ~0.5 ^◦^C decrease (Fig. 5A). Next, as TAAR1 agonists reportedly exert an inhibitory effect on locomotion [42], we examined baseline locomotion in both genotypes with and without SEP-856 (10 mg/kg) pretreatment. We observed a clear decrease of baseline locomotion exclusively in WT, but not in TAAR1-KO, mice (Fig. 5B).

After confirming two distinct yet TAAR1 dependent physiological impacts of SEP-856, we continued by investigating TAAR1 dependent effects using different clinically relevant behavioral models related to potential antipsychotic efficacy. Specifically, we first tested if SEP-856 mediated a TAAR1 dependent effect on pre-pulse inhibition (PPI). RM-two way ANOVA showed a significant SEP-856 (10 mg/kg) effect at the highest pulse sound (12 dB) (Treatment: F_(1, 34)_ = 6.109, *p* = 0.0186; Fig. 5C). Post-hoc analysis revealed a statistically significant increase in PPI with SEP-856 in WT mice (*p* = 0.0045), a phenomenon, which was abolished in TAAR1-KO mice (Fig. 5C). To consolidate the role of TAAR1 in this phenomenon, we proceeded to disrupt baseline PPI using the NMDA receptor antagonist MK-801 (0.4 mg/kg). RM-two way ANOVA showed a significant treatment effect at the highest pulse sound (12 dB) (Treatment: F_(2, 42)_ = 35.36, *p* < 0.0001; Fig. 5D). Post-hoc test revealed a significant reversal of MK-801 induced PPI disruption in WT (*p* = 0.0114), but not TAAR1-KO mice with SEP-856 (10 mg/kg) at the highest intensity of pre-pulse (Fig. 5D). Next, we investigated whether SEP-856 was capable of decreasing MK-801 induced hyperactivity. MK-801 (0.4 mg/kg) treated mice displayed a significant increase in traveled distance, whereas pre-treatment with SEP-856 (10 mg/kg) was able to diminish this behavior, restoring it to the level observed in animals treated with vehicle (VEH) alone (Treatment: F_(2, 24)_ = 32.5, *p* < 0.0001; Fig. 5E). Post-hoc analysis showed that SEP-856 was able to suppress MK-801 hyperactivity in both WT (*p* = 0.0015) and TAAR1-KO mice (*p* = 0.0007). No significant genotype or sex differences (Supplementary Fig. 8) were observed between MK-801 or SEP-856 responses.

Finally, it is well established that D_2_ receptor antagonists block d-amphetamine induced hyperlocomotion in vivo, a prototypical model of antipsychotic activity. For this reason, and since TAAR1 agonists can inhibit dopaminergic cell signaling [42] presumably due to GIRK channel activation, we investigated whether SEP-856 can blunt the locomotor stimulation induced by d-amphetamine (5 mg/kg, Supplementary Fig. 6A). RM-two way ANOVA showed a significant effect of amphetamine treatment to increase locomotion (Treatment: F_(2,44)_ = 81, *p* < 0.0001). However, SEP-856 (10 mg/kg) failed to suppress d-amphetamine induced hyperactivity in both WT and TAAR1-KO mice. Conversely, modest potentiation of d-amphetamine induced locomotion was observed in TAAR1-KO mice compared with WT mice. Additionally, certain TAAR1 agonists have been shown to counteract hyperlocomotion induced by other hyperdopaminergic stimulants such as cocaine [42]. Therefore, we evaluated whether SEP-856 (10 mg/kg) pre-treatment could decrease cocaine- (20 mg/kg) induced locomotion stimulation (Treatment: F_(2,22)_ = 44, *p* < 0.0001). By analogy to our earlier observations using d-amphetamine, we did not detect any significant inhibition of cocaine-mediated hyperlocomotion with SEP-856 pre-treatment in either genotype (Supplementary Fig. 6B).

## Discussion

### Main discussion

SEP-856 is attracting considerable interest owing to its clinically validated antipsychotic properties paired with a low affinity for the D_2_ receptor [10, 12]. Furthermore, it displays pronounced activity at TAAR1 [10, 43]. while sharing the 5-HT_1A_ partial agonism of several other antipsychotics [10]. However, in the initial study which introduced the compound, no studies in TAAR1 deficient mice were performed [10] and the G-protein coupling profile of SEP-856 at TAAR1 and 5HT1_A_ receptors were not explored. Herein, we demonstrate that the antipsychotic-like behavioral profile of SEP-856 involves TAAR1, although it is not fully dependent on this receptor. Further, employing a codon-optimized TAAR1 construct together with a 5HT_1A_ receptor construct, we also evaluated the G-protein coupling profiles and GIRK activation capacity of SEP-856 at both receptors in vitro.

The generation of a successful assay system to investigate TAAR1 activation and Gα protein dependent TAAR1 signaling was pivotal for the current work. Indeed, it has been suggested that TAAR1 displays G-protein coupling promiscuity, with signaling via multiple Gα-protein couplings reported [35, 44]. Here, using our novel assay system, we show that SEP-856 displays full agonist properties in TAAR1 coupling to Gα_s_ but does not induce recruitment of Gα_i_, Gα_q_ and Gα_12_. Prominent Gα_s_ coupling paired with a pEC_50_ of 6.49 for β-PEA matches well with potencies described by Lindemann et al. [45] (pEC_50_: 6.52), Navarro et al. [44] (pEC_50_: 6.79) and Borowski et al. [46] (pEC_50_: 6.49), indicating the assay is accurate and sensitive enough to allow conclusions about TAAR1 pharmacology to be drawn. Furthermore, the rank-order of potencies in this assay closely matches those reported in previous reports, with β-PEA and p-Tyramine ranking as high potency agonists compared with dopamine and norepinephrine. Importantly, we were able to utilize the same hTAAR1 construct (minus a SmBiT tag) in *Xenopus* oocytes for electrophysiological studies. Our results show that SEP-856 is able to induce TAAR1 dependent GIRK channel activation [20]. Curiously, SEP-856 appears to act as a partial agonist towards GIRK channel activation despite acting as a full agonist in Gα_s_ recruitment assays and cAMP accumulation assays [10]. This unique feature is likely to play a role in its in vivo effects but further studies are required to elucidate the functional consequences of cAMP/PKA activation combined with ion channel mediated inhibition.

The cellular localization of a receptor is understood to play a role in the outcome of its signaling event [47]. Importantly, unlike most GPCRs, TAAR1 is mainly localized intracellularly [33, 48]. Our own data corroborates this finding, with the most abundant colocalization of TAAR1 observed together with the ER resident protein Calrectulin. Additionally, TAAR1 colocalization with the Golgi marker BGAL4-T1 but not the trans-Golgi marker TGN38 supports the limited forward trafficking of TAAR1 to the surface plasma membrane. Nevertheless, we observed TAAR1 activation by cell membrane-impermeable compounds such as dopamine and norepinephrine. Therefore, we showed that WT-TA1 (TAAR1 lacking N-glycosylation sites or additional surface localization boosters) can be detected on the cell surface plasma membrane in low yields using flow cytometry. This finding is supported by another recent study [36], which described plasma membrane localization of TAAR1 in a thyroid cell line. Collectively this data suggests that SEP-856 can signal through surface plasma membrane TAAR1 coupled to Gα_s_.

Our behavioral studies uncovered two distinct physiological responses to SEP-856 which help explain its TAAR1 agonist properties in vivo. First, we showed that SEP-856, similar to other TAAR1 agonists, decreases core body temperature presumably reflecting TAAR1 expression in the neurons of the medial optic nucleus [41]. Next, we demonstrated that SEP-856 exerts a strong inhibitory effect on baseline locomotion in WT, but not TAAR1-KO mice. Considering that TAAR1 is expressed in both dopamine and glutamate neurons [22, 41], we questioned if SEP-856 effects on hyperdopaminergic (d-amphetamine, cocaine) and hypoglutamatergic (MK-801) drug induced hyperlocomotion are mediated by TAAR1. Interestingly, SEP-856 hinders MK-801 induced hyperactivity regardless of the genotype but fails to counteract the effects of dopamine-releasing agents (amphetamine and cocaine) on locomotion. Finally, we demonstrate that the ability of SEP-856 to increase sensorimotor gating (PPI) is reliant on TAAR1. Dedic et al. [10] showed modulation of PPI by SEP-856 at doses of 3, 10 or 30 mg/kg, which is in agreement with our data at 10 mg/kg. Furthermore, we extend on this behavioral finding by showing that SEP-856 pre-treatment counteracts MK-801 induced baseline PPI disruption exclusively in WT but not TAAR1-KO mice. This model, even if still with limitations, is more relevant to putative antipsychotic properties than baseline PPI. Additionally, the selective TAAR1 agonist o-phenyliodotyramine was recently shown to increase PPI in WT but not in TAAR1-KO mice [41] which supports the notion of agonism at this receptor directly influencing sensorimotor gating. Pre-pulse inhibition remains one of the primary behavioral tools to study potential antipsychotic like effects of compounds. Therefore our data underpins the relevance of TAAR1 agonism to antipsychotic-like properties, at least in regards to a reinforcement of the ability to filter out irrelevant information.

5-HT_1A_ receptors are targeted by numerous psychoactive molecules such as psychedelics (LSD, psilocin) anxiolytic agents (Buspirone) female hypoactive sexual desire disorder medications (Flibanserin) and antidepressants (Vilazodone). The physiological actions of these 5-HT_1A_ ligands are likely partially dependent on selective targeting of 5-HT_1A_ in different brain locations as a result of differential G-protein coupling profiles [49]. Even though 5-HT_1A_ is typically described as Gα_i/o_-coupled, promiscuous Gα-protein coupling has been shown with coupling to both Gα_i_ and Gα_q_ proteins [37, 38]. Here, we report that SEP-856 behaves as a low-potency partial agonist for 5-HT_1A_, able to recruit Gα_i_ and Gα_q_ equally well, and provide evidence for Gα_s_ recruitment. This relatively poor G-protein recruitment induced via SEP-856 was also seen with β-arrestin recruitment, without any obvious signal bias between the two observed when comparing the response to serotonin. Additionally, we show that despite low-potency partial agonism, SEP-856 can act as a weak antagonist on the 5-HT_1A_ receptor. Moreover, SEP-856’s 5-HT_1A_ partial agonism is not only observable with Gα-and β-Arrestin coupling but also in Gβγ derived GIRK activation. Interestingly, the pEC_50_ fold difference between serotonin and SEP-856 in these assays varied (1.25 × 5-HT vs SEP-856, Gα_i_ and 1.70 × 5-HT vs SEP-856, GIRK) indicative of a potentially lower efficacy of SEP-856 to activate GIRK channels than to recruit Gα proteins.

Our results from both Gα recruitment and GIRK activation assays indicate a lower potency of SEP-856 at the 5-HT_1A_ receptor than Dedic et al. [10] (pEC_50_: 5.64 cAMP inhibition vs. 4.76 Gα_i_ recruitment, 4.49 β-arrestin recruitment and 5.08 GIRK activation). Likewise, while our TAAR1 G-protein recruitment assay data largely agrees with existing observations for β-PEA potency on the receptor, the described potency of SEP-856 here (pEC_50_: 6.08) is slightly lower than described by Dedic et al. (pEC_50_: 6.85) and another cAMP accumulation assay published more recently [50]. These differences can potentially be explained due to the differences in functional assays employed here, as second messenger assays are typically very sensitive and are prone to signal amplification. Different signaling assays are known to elicit different responses from ligands which may be a consequence of ligand bias or a limitation of the assay itself [51]. Likewise, the degree of G-protein recruitment required for second messenger generation and saturation is not well described. However, the profile of SEP-856 as a 5-HT_1A_ receptor low-potency partial agonist is validated and expanded on in this study. It is unclear to what extent SEP-856 acts in vivo through 5-HT_1A_ receptors in view of the relatively low to modest potencies described here. The ability of SEP-856 to suppress MK-801 induced hyperactivity, even following TAAR1 deletion, indicates a possible involvement of 5-HT_1A_ receptors in SEP-856 mediated antipsychotic-like efficacy. This is supported by the dense expression of 5-HT_1A_ receptor on cortical and hippocampal neurons [52] and the effect of 5-HT_1A_ receptor antagonists on hypoglutamatergic states [53, 54]. Likewise, we find that the potent 5-HT_1A_ antagonist NAN-190 (Fig. 3D) is able to counteract MK-801 induced hyperactivity in both genotypes (Supplementary Fig. 7). Furthermore, TAAR1 activation is known to increase potencies of 5-HT_1A_ targeting drugs in 5-HT neurons, which may reflect an increased response in vivo that is not presently investigated in vitro [42]. Low potency partial agonism at the 5-HT_1A_ receptor is a feature of several antipsychotics such as aripiprazole and therefore cannot be ignored in the functional profile of SEP-856 [9, 29]. Also, a recent in silico perspective utilizing molecular dynamics simulations supports functions at both TAAR1 and the 5-HT_1A_ receptor [55].

Finally, in agreement with the initial SEP-856 study, we show that this ligand exhibits partial D_2_ receptor agonism and antagonism in vitro, albeit with very low potency and efficacy, making this property unlikely to play any meaningful role in vivo. Also, SEP-856 maintains its weak D_2_ receptor partial agonist properties with regards to  Gβγ dependent GIRK activation, in line with the previously described low efficacy of this drug at the D_2_ receptor. In vivo, SEP-856 has not been previously evaluated using the d-amphetamine-induced hyperlocomotion in WT and TAAR1-KO mice [56], a well-established model for evaluating D_2_ antagonists and antipsychotics. Similarly, other TAAR1 agonists have been reported to display inhibition of cocaine mediated hyperactivity [23, 42] and L-DOPA-induced motor sensitization [53]. However, it is unclear if all TAAR1 agonists are capable of suppressing hyperdopaminergic psychostimulant-mediated locomotion. For example, RO5073012, a TAAR1 partial agonist with low intrinsic efficacy, failed to reduce d-amphetamine hyperlocomotion in WT mice [57]. In the same study, it is reported that d-amphetamine fails to trigger hyperlocomotion in transgenic TAAR1-overexpressing mice. However, RO5073012 restored the sensitivity of TAAR1-overexpressing mice to d-amphetamine [57]. Additionally, TAAR1-KO mice display an enhanced sensitivity to monoamine releasing agents [19, 21, 22, 58]. Therefore, it was important to assess the effectiveness of SEP-856 using both the d-amphetamine and cocaine behavioral challenges to not only exclude any potential classical D_2_ antagonism but also expand on the limited data with TAAR1 agonists using hyperdopaminergic stimulants. In our study, we demonstrate that SEP-856, despite acting as a full TAAR1 agonist, does not reduce d-amphetamine or cocaine induced hyperlocomotion in either WT or TAAR1-KO mice. Conversely, we noticed a small potentiation of amphetamine hyperlocomotion in TAAR1-KO mice with SEP-856 treatment. TAAR1-KO mice appear to sensitize faster to amphetamine than WT mice [59] and it is possible SEP-856 accelerates this process through as of yet, an unknown mechanism. Nonetheless, the inability of SEP-856 to inhibit d-amphetamine effects in vivo supports a non-potent D_2_ antagonistic profile of the drug.

#### Limitations and future outlook

While our study overcomes several challenges of TAAR1 pharmacology, several others justify further studies. Since TAAR1 appears to be largely intracellular and trace amines have been shown to readily cross cellular membranes [60], it will be important to study signaling at these locations separately. ER TAAR1 signaling may be exclusive to Gα_13_ as has been recently suggested for amphetamine [35] and further work is needed to separate SEP-856 signaling outcomes in different cellular compartments. Additionally, while we have explored the molecular pharmacology of SEP-856 on the 5-HT_1A_ receptor in detail here, our analysis did not conclusively address the role 5-HT_1A_ receptor-dependent effects of SEP-856 on behavior. Future investigations would ideally involve 5-HT_1A_ receptor-KO mice since 5-HT_1A_ receptor antagonists themselves alter relevant behavioral readouts (Supplementary Fig. 7), making 5-HT_1A_ receptor pharmacological intervention of SEP-856 studies difficult to interpret. In their recent review [11], Dedic et al. also point to the possible contribution of other serotonergic receptors (namely 5-HT_7_ and 5-HT_1B_) to the clinical efficacy of SEP-856 which showed modest to poor potencies for the compound. These receptors were not explored in this paper but warrant additional studies.

Several behavioral readouts have been developed to screen for, and study antipsychotic like actions of compounds. In this paper, we have used two of the arguably most established assays, sensorimotor gating (PPI) and inhibition of hyperlocomotion induced by psychostimulants. Since SEP-856 is able to diminish MK-801 hyperactivity (this paper) and PCP hyperactivity [10], but not d-amphetamine or cocaine induced hyperactivity, the effectiveness of SEP-856 as an antipsychotic justifies further behavioral studies which may also aid in classifying the behavioral phenotype of novel, non-dopaminergic antipsychotics. Interestingly, atypical antipsychotics with 5-HT_2A_ receptor antagonism such as clozapine appear to display greater antagonism of hyperactivity/stereotypy exerted by PCP than d-amphetamine [61–63].

In conclusion, while certain other (still to be unveiled) mechanisms of action of SEP-856 may be involved in its clinical efficacy in schizophrenia, the present study sheds further light on the nature of its molecular interactions with TAAR1 and 5-HT_1A_ receptor sites, and supports their likely collective roles in the expression of its antipsychotic and other functional actions. In view of the originality and therapeutic effectiveness of SEP-856, which may represent true progress in the management of schizophrenia and other psychotic disorders, additional studies are warranted to further clarify exactly how it exerts its actions.

## Supplementary information

Supplementary Methods and Figures
