# Correction To: TAAR1 dependent and independent actions of the potential antipsychotic and dual TAAR1/5-HT_1A_ receptor agonist SEP- 363856

**DOI:** 10.1038/s41386-022-01504-0

**Published:** 2022-11-29

**Authors:** Marcus Saarinen, Ioannis Mantas, Ivana Flais, Richard Ågren, Kristoffer Sahlholm, Mark J. Millan, Per Svenningsson

**Affiliations:** 1 Department of Clinical Neuroscience, Karolinska Institutet, Stockholm, Sweden; 2 Basic and Clinical Neuroscience, King’s College London, London, UK; 3 Department of Neuroscience, Karolinska Institutet, Stockholm, Sweden; 4 Department of Integrative Medical Biology, Wallenberg Centre for Molecular Medicine, Umeå University, Umeå, Sweden; 5 Neuroinflammation Therapeutic Area, Institut de Recherches Servier, Centre de Recherches de Croissy, Paris, France and Institute of Neuroscience and Psychology, College of Medicine, Vet and Life Sciences, Glasgow University, Scotland, Glasgow, UK

**Keywords:** Cellular neuroscience, Schizophrenia

Correction to: *Neuropsychopharmacology* 10.1038/s41386-022-01421-2, published online 13 September 2022

The technical name of the compound studied in this article was incorrectly referred to as SEP-383856 in the title and the abstract and has now been corrected to SEP-363856. None of the scientific conclusions in this paper are affected by this correction. The original article has been corrected.

