# Supplementary material for: TAAR1 dependent and independent actions of the potential antipsychotic and dual TAAR1/5-HT1A receptor agonist SEP-363856
Source: Neuropsychopharmacology. 2022 Sep 13;47(13):2319–29. doi: 10.1038/s41386-022-01421-2 (PMC9630386; doi:10.1038/s41386-022-01421-2)
Supplement: Supplementary file 1 — Supplementary Methods and Figures [file 41386_2022_1421_MOESM1_ESM.pdf]

## Supplementary Methods

***Cell culture:*** All cell culture reagents were obtained through Thermo Fischer Scientific unless otherwise stated. Expi293F cells were kindly gifted by Ilana Kotliar (Rockefeller University, NY,USA). Cells were cultured according to manufacturers guidelines. Briefly, cells were maintained in Expi293 expression medium using 125mL polycarbonate vented Erlenmeyer shake flasks (Corning) with constant orbital shaking at 150RPM (Heidolph, Germany). Cells were maintained in a humidified environment with 37°C and 8% CO2. HEK293T cells were purchased from ATCC, and were maintained in Dulbeccos modified eagle medium (DMEM) supplemented with 1% penicillin-streptomycin, 1mM sodium pyruvate, minimal essential medium non-essential amino acids, HEPES buffer and GlutaMax. HEK293T cells were grown in 100mm tissue culture dishes (Sarstedt) at 37°C, 5% CO2 and a humidified environment.

***Animals:***

Male and female (2-5 months old) WT and TAAR1-KO animals[1] were housed in temperature- and humidity-controlled rooms (20°C, 53% humidity) with a 12 hour light/dark cycle and access to food pellets and water *ad libitum*.

***Drugs:***

For mammalian cell culture experiments all compounds used for in vitro studies were dissolved in dPBS (Thermo Fischer Scientific) containing 100µM ascorbic acid (Sigma-Aldrich). Norepinephrine (+/-)-bitartrate salt, dopamine hydrochloride, β-PEA, p-Tyramine were obtained through Merck. For animal studies, SEP-856 (MedChemExpress) was dissolved in solution containing 20% (2-Hydroxypropyl)-β-cyclodextrin (Sigma-Aldrich) in saline. D-amphetamine and MK-801 (Sigma-Aldrich)

were dissolved in saline solution. NAN-190 (Tocris) was dissolved in solution containing 10% dimethyl sulfoxide (Sigma Aldrich) in saline.

***Behavioral test:***

Body temperature measurement: Core temperature was determined by a rectal thermoprobe in two different days. The first day the mice were treated with vehicle 30 minutes before temperature measurement. The second day the mice were administered SEP-856 (10 mg/kg, per os (p.o.)) 30 minutes before core temperature measurements.

Pre-pulse inhibition (PPI): The mice were subjected to PPI at three separate days. The first day the mice were treated with vehicle (p.o.) 30 minutes before testing. The second day the mice were administered with MK-801 (0.4mg/kg). On the third day, the mice were administered SEP-856 (10 mg/kg, p.o.) 30 minutes before being administered MK-801 (0.4mg/kg). After 30 minutes, the PPI test commenced. For PPI testing we used two startle chambers (San Diego Instruments, San Diego, Calif., USA). Each chamber contained a Plexiglas cylinder attached on a platform and a loudspeaker that produced both a continuous background noise of 65 dB and the different acoustic stimuli. Mouse startle responses caused vibrations of the cylinder, which were transformed to analog signals by a piezoelectric transducer under the platform. Calibrations were performed on the chambers before every experiment. Each test session started with a 5-min background noise (65 dB white noise) habituation period. The background noise continued throughout the test session. The habituation period followed by four blocks of trials, with an average interval of 12 seconds. The first and the sixth block consisted of five Startle (a 40-ms 120 dB burst) trials. Four trial types were presented during the second and the third block in a pseudo-randomized sequence with ten of each trial type per block. Trial types contained a Startle and three separate pre-pulse trials in which 20-ms long prepulses

of different intensities (68, 71, or 77 dB) preceded the startle stimulus by a 100 ms interval. The test session lasted for a total of 23 min and contained 60 trials.

Open field test (OFT): For the assessment of SEP-856 on baseline locomotion, mice were treated per os (p.o.) with vehicle or SEP-856 (10 mg/kg) 30 minutes before the test. Then the mice were subjected to OFT for 30 minutes. For the psychostimulant-induced hyperlocomotion tests, the mice were subjected to OFT on three separate days. The first day the mice were treated with vehicle (p.o. or intra peritoneal (i.p.)) 30 minutes and immediately before OFT. The second day the mice were treated with vehicle (p.o. or i.p.) 30 minutes before testing and MK-801 (0.4 mg/kg, i.p.), d-amphetamine (5 mg/kg, i.p.) or cocaine hydrochloride (20mg/kg) immediately before OFT. The third day the mice were pretreated with SEP-856 (10 mg/kg, p.o.) or NAN-190 (3 mg/kg, i.p.) 30 minutes and 10 minutes before testing, respectively. Subsequently, the mice were treated with MK-801 (0.4 mg/kg, i.p.), d-amphetamine (5 mg/kg, i.p.) or cocaine hydrochloride (20mg/kg) immediately before OFT. Mice treated with MK-801 or d-amphetamine were recorded for 60 minutes, while mice treated with cocaine were recorded for 30 minutes. The OFT arena was 46 × 46 $cm^2$ wide with grey floor and walls. The arena was cleaned with 70% ethanol after each test session to eliminate olfactory cues. Video tracking was performed using a video camera mounted in the ceiling and analyzed by EthoVision XT11.5 (Noldus) software

***Molecular biology:*** cDNA encoding dopamine D2 (D2R; long isoform) and serotonin 1A (5-HT1AR) receptors and GIRK1/4 (GenScript) were in pXOOM [2]. $D_2R$, 5-$HT_{1A}$, and GIRK1/4 were linearized using the XhoI, XbaI, and NotI restriction enzymes, respectively, followed by in vitro transcription using the T7 mMessage mMachine kit (Ambion, Austin, TX). cRNA concentration and purity were determined by

spectrophotometry. A mammalian, codon-optimized (GenSmart, GenScript) human TAAR1 construct was generated with triple HA-tags on the N-terminus, and a SmBiT linker on the C-terminus (GenScript), with or without an additional 9 amino acids from the N-terminus of the beta2-adrenergic-receptor and subcloned into pcDNA 3.1+. The same (β-TA1) insert was subcloned into pXOOM without the C-terminal SmBiT tag and was used for in vitro cRNA transcription for oocyte injection following linearization with XhoI. The human $5HT_{1A}$ and $D_2$ CDS's were separately subcloned via GenScript into an N-terminal mGluR5 signal peptide-HA tag construct backbone, with a SmBiT tag on the C-terminus (kind gift of Drs. Franscisco Ciruela and Xavier Morato). Mini-G proteins were LgBiT tagged on the N-terminus, synthesized and generated at GenScript according to published sequences [3, 4], without polyhistidine tags. β-Arrestin2 constructs were generated by subcloning the β-Arrestin2 CDS into either N or C-terminal LgBiT containing donor plasmids (Promega). See Supplementary figure 1 for more details. mCherry tagged localization markers were gifts from Michael Davidson (Addgene plasmids #55008, #55052, #55145, #55006, # 55102)

***Oocyte preparation and electrophysiology****:* Oocytes from the African clawed toad, *Xenopus laevis*, were isolated as described previously[5]. The procedure was approved by the Swedish National Board for Laboratory Animals and the Animal Welfare Ethical Committee in Stockholm (approval number 686–2021). After one day of incubation at 12°C, oocytes were injected with 50 nl containing 0.2 ng D2R or 5-HT1AR cRNA, and 40 pg of each G-protein inward rectifying potassium channel (GIRK) 1 and GIRK4 cRNA using the Nanoject III (Drummond Scientific, Broomall, PA). For experiments with TAAR1, 1 ng of TAAR1 cRNA +/- 1 ng of GNAS (encoding Gαs) cRNA were injected along with GIRK1/4 cRNA (40 pg of each subunit). Injected cells were

incubated for 6 days at 12°C in an aqueous solution containing 88 mM NaCl, 1 mM KCl, 2.4 mM $NaHCO_3$, 15 mM HEPES, 0.33 mM $Ca(NO_3)_2$, 0.41 mM $CaCl_2$, 0.92 mM $MgSO_4$, 2.5 mM sodium pyruvate, 25 U/ml penicillin, and 25 µg/ml streptomycin, adjusted to pH 7.6 using NaOH. Electrophysiological recordings were performed at 22°C using the semi-automated two-electrode voltage-clamp OpusXpress 6000A (Molecular Devices, San Jose, CA) [6]. Continuous perfusion was maintained at 1 ml/min. Data were acquired at membrane potentials of -80 mV and sampled at 156 Hz using the OpusXpress 1.10.42 (Molecular Devices, CA) software. To increase the inward rectifier potassium channel current at negative potentials, a high-potassium extracellular perfusion buffer was used (64 mM NaCl, 25 mM KCl, 0.8 mM MgCl2, 0.4 mM CaCl2, 15 mM HEPES, and 1 mM ascorbic acid, adjusted to pH 7.4). Ascorbic acid was included to prevent the spontaneous oxidation of dopamine. Dopamine and serotonin (Sigma-Aldrich, St. Louis, MO) were prepared fresh and dissolved directly in 25 mM KCl recording buffer.

***Transfections:*** Expi293F cells were diluted to 3e6 cells/mL in 2.5mL of Expi293 Media in 6-well plates (Sarstedt). Cells were transfected with 2.25µg of receptor construct and 0.25µg mini G-protein construct with a 1:3.2 ratio of DNA:Expifectamine. The following day, transfection enhancers were added and cells were used after 48h of transfection. For flow cytometric analysis, receptors were transfected without co-transfection of mini G-proteins. HEK293T cells were seeded in 35mm imaging dishes (Ibidi) with 300.000 cells/dish in 2mL of supplemented DMEM media. The following day, cells were transfected with 1µg of receptor construct + 1µg of mCherry tagged cell compartment marker, with linear polyethylenimine (Polysciences, Inc) used as a transfection reagent at a ratio of 1:4 of DNA:PEI. Cells were used for imaging 24h after transfection.

***Confocal imaging microscopy:*** For imaging, HEK293T cells transfected in Ibidi 35mm imaging dishes were fixed with 4% formaldehyde at room temperature (RT) for 15 minutes. Following fixation, cells were permeabilized using 0.1% Triton-X 100 (Sigma) in PBS for 10 minutes at RT. Cells were blocked in 4% FBS diluted in PBS for 1h at RT, after which cells were stained overnight with anti-HA epitope antibodies (Biolegend 16B12, 1:200) at 4˚C, followed by addition of Alexa Flor 488 conjugated anti-mouse secondaries (Invitrogen A-21202, 1:500) for 2h at RT. Both antibody incubation steps were performed in 4% FBS in PBS, and necessary washing steps using PBS were taken between all steps. Cells were then stained with 300mM DAPI for 5min at RT, washed, and mounted with Ibidi mounting solution. Cells were then imaged using a laser scanning confocal microscope (Zeiss 880 LSM, Germany) using 405nm, 458nm and 561nm lasers and an 63x/1.4 M27 OIL DIC Plan-Apochromat objective with a PMT detector configurated for DAPI, Alexa Fluor 488 and mCherry emission. Images were analyzed using Fiji.

***Flow cytometry:*** For flow cytometry analysis, Expi293F cells were collected and spun down for 10min, at 100g in a pre-chilled centrifuge. Cells were resuspended in PBS/2% FCS and labeled with a live-death dye (1:1000, eBioscience, Thermo Fischer) for 10 minutes. Cells were fixed with 4% formaldehyde for 15min on ice, and afterwards stained with PE conjugated anti-HA antibodies (Biolegend 16B12, 1:200) for 2h at RT. Washing with PBS/2% FCS was performed between all steps. Data were acquired on an LSR Fortessa Flow Cytometer (BD Biosciences, Stockholm, Sweden) and analyzed with FlowJo software v.10 (BD).

***G-protein recruitment***: 48h after transfection with min G-proteins/βArr2 and receptors, cells were diluted to a total volume of 10mL in dPBS and 10μM of coelenterazine-h (NanoLight). Cells were then reseeded in solid white, 96 well plates (Corning) with 90 μL of cell suspension/well. Each plate was read using luminescence detection mode (SPARK 10M, Tecan, Sweden) before addition of compounds to ensure signal stability. 10 μL of compounds diluted in 100 μM ascorbic acid were added, and luminescence from each well was read for 5 minutes, with 100ms read time/well. Due to a relatively short time difference between column stimulation because of manual pipetting between columns, data captured at each relative stimulation peak was used for further data analysis

***Data analysis:*** For mammalian cell culture experiments and oocyte voltage clamp electrophysiology recording derived concentration-response relationships were analyzed by fitting three parameter (agonist or inhibitor) concentration response curves (Hill slope =1, -1 respectively) in GraphPad Prism 9 (GraphPad Software, San Diego, CA). For each concentration of SEP-856, the evoked GIRK current response was normalized to the response evoked by 100 nM 5-HT (in experiments with the 5-$HT_{1A}$ receptor), 1 μM dopamine (in experiments with the $D_2$ receptor) or 1mM p-Tyramine (in experiments with TAAR1) in the same oocyte. For animal studies, statistical analysis was carried out by one-/two-way repeated-measures analysis of variance (RM-ANOVA), followed by Sidak post hoc multiple comparison test. Both statistical analysis and data plotting was performed by using GraphPad Prism 8.0 and presented as mean ±SEM of the number of subjects/samples per group.

## Supplementary figures

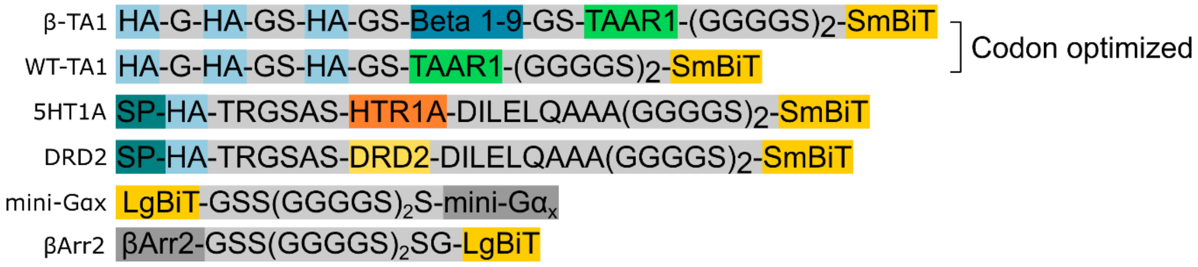


**Supplementary Figure 1: Construct overview**

The mini-G-proteins used in this study are $G_s$393, $G_{si}$143, $G_{sq}$70 and $G_sG_{12}$ for $G\alpha_s$, $G\alpha_i$, $G\alpha_q$ and $G\alpha_{12}$ recruitment, respectively[3, 4]

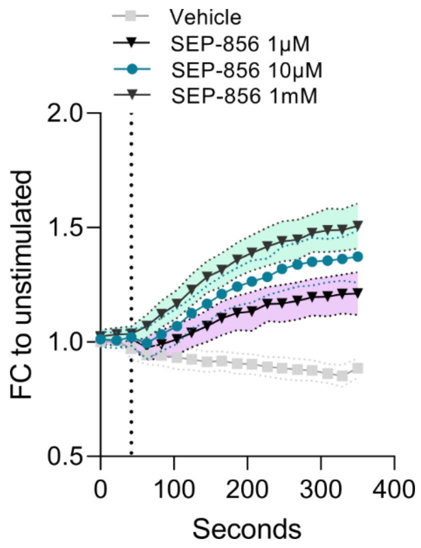


**Supplementary Figure 2: TAAR1 $G\alpha_s$ recruitment via SEP-856**

Shown is a plot from various stimulating concentrations of SEP-856 on WT-TA1 $G\alpha_s$ recruitment over time, with a saturating concentration (1mM, same as Figure 1A) for comparison. N=3 for all, error bars represent S.E.M.

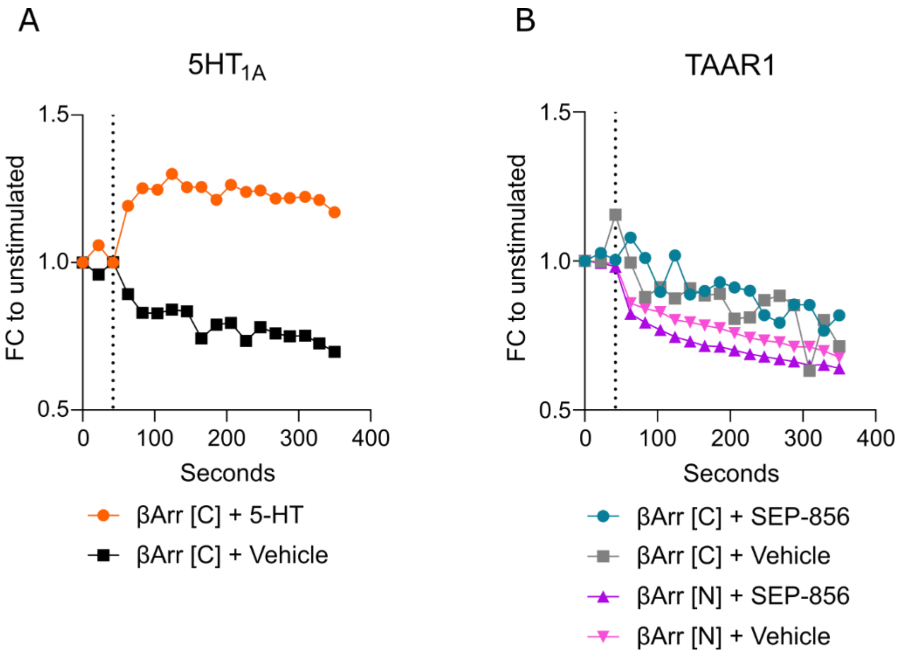


**Supplementary Figure 3: TAAR1 β-Arrestin recruitment**

Representative traces of β-Arrestin2 constructs tagged with either a C-terminal (βArr[C]) or N-terminal (βArr[N]) LgBiT co-transfected with SmBiT tagged $5HT_{1A}$ (A) or WT-TAAR1 (B). Dotted line indicated addition of 1mM of either 5-HT (A) or SEP-856 (B).

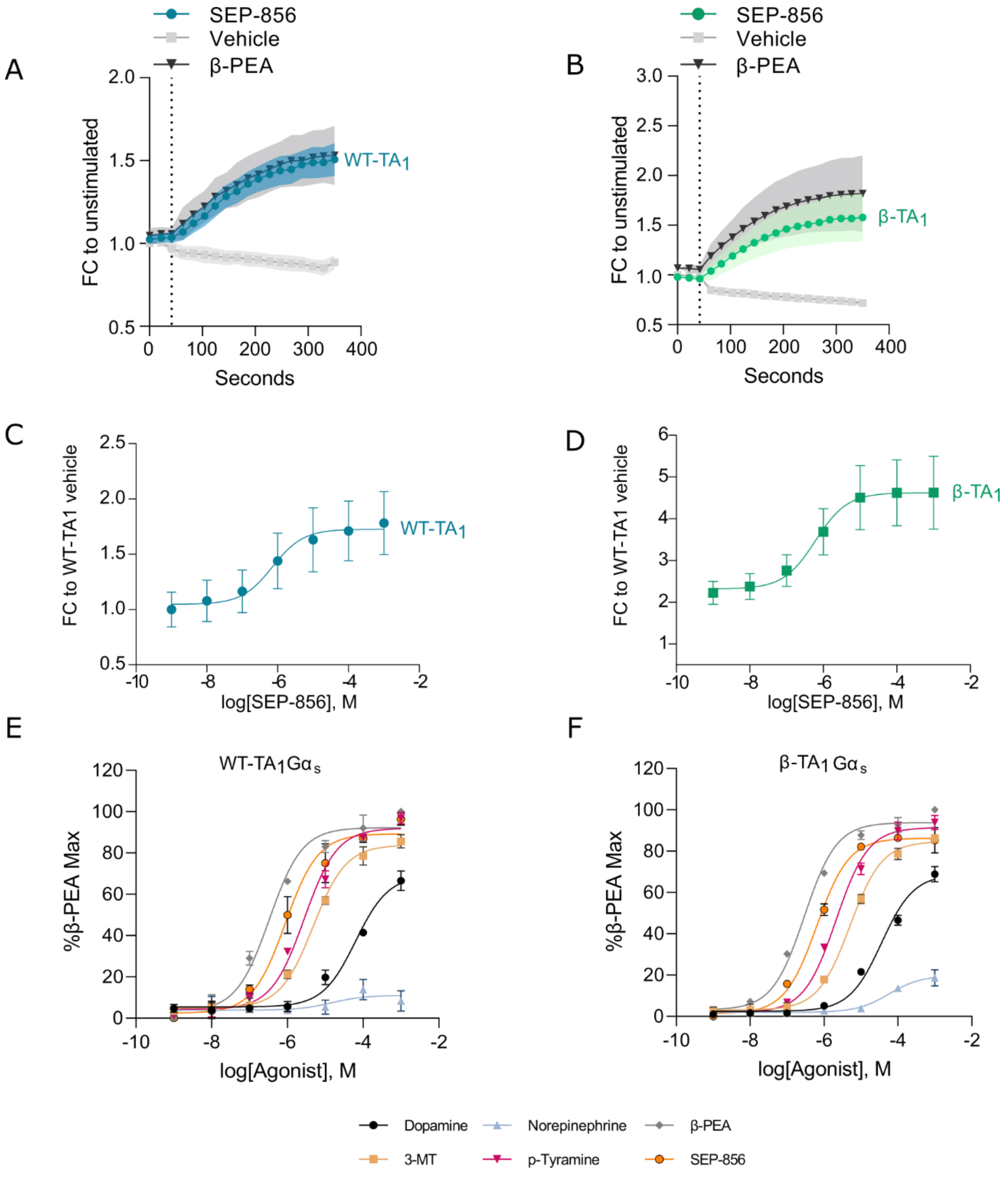


**Supplementary Figure 4: Comparison of TAAR1 constructs**

Left: WT-TA1 Right: β-TA1 (A-B) time course of luminescence responses for the two TAAR1 constructs, each normalized to their pre-treatment state and expressed as fold change (FC). (C-D) Comparison of maximal and baseline luminescence calculated as a fold change relative to the WT baseline luminescence. (E-F) concentration-response graphs of both constructs with various TAAR1 agonists for comparison. All results shown are from at least three independent plates ran in duplicates.

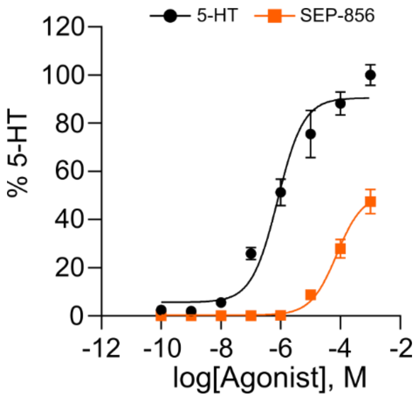


**Supplementary Figure 5: 5-HT$_{1A}$ - G$\alpha_s$ concentration response-curve**

G-protein recruitment (G$\alpha_S$) was determined for both 5-HT and SEP-856 induced stimulation. N=3, pEC$_{50}$ 5-HT:6.10 ±0.10

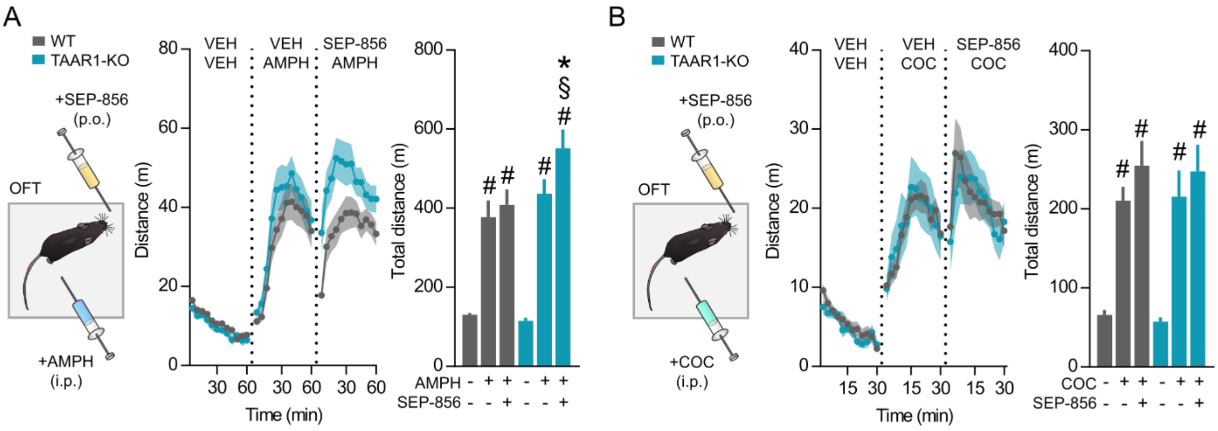


**Supplementary Figure 6: Effect of SEP-856 on d-amphetamine and cocaine induced hyperlocomotion**

(A) SEP-856 (10mg/kg) was evaluated using the OFT to assess potential inhibition of d-amphetamine (5mg/kg) mediated hyperlocomotion in WT (n=14) and TAAR1-KO (n=10) mice (Treatment: $F_{(2, 44)} = 81$, $p<0.0001$; Genotype x Treatment: $F_{(2, 44)} = 3.553$, $p=0.0371$; *, $p<0.05$, WT vs TAAR1-KO; #, $p<0.0001$ Vehicle - Vehicle vs Vehicle-D-amphetamine, §$p<0.05$ Vehicle- D-amphetamine vs SEP-856-D-amphetamine, Sidak's post-hoc). Time bins shown represent 5-minute intervals.

(B) SEP-856 (10mg/kg) was similarly used to evaluate any influence on cocaine (20mg/kg) mediated hyperlocomotion in WT (n=7) or TAAR1-KO (n=7) mice (Treatment: $F_{(2, 22)} = 43.8$, $p<0.0001$; Genotype x Treatment: $F_{(2, 22)} = 0.06$, $p=0.9419$; *, $p<0.05$, #, $p<0.0001$ Vehicle - Vehicle vs Vehicle-Cocaine, Sidak's post-hoc). Time bins shown represent 2.5-minute intervals.

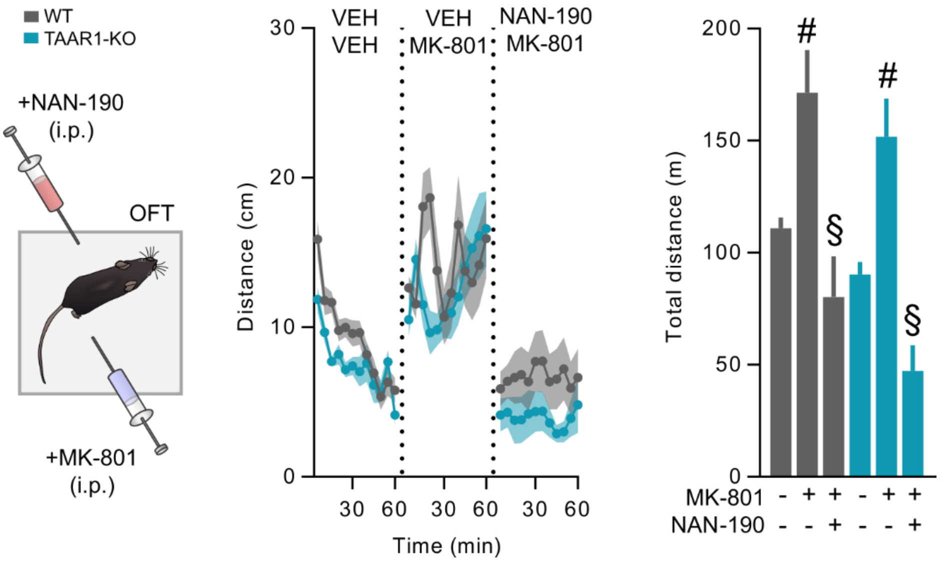


**Supplementary Figure 7: NAN-190 actions on MK- 801-induced hyperlocomotion**

Effect of the $5HT_{1A}$ antagonist NAN-190 (3mg/kg) on MK-801 (0.4mg/kg) induced hyperlocomotion was assessed using the OFT (WT n=11, TAAR1-KO n=9) (Treatment: $F_{(2, 36)} = 31.75$, $p<0.0001$; Genotype x Treatment: $F_{(2, 22)} = 0.1814$, $p=0.98349$; *, $p<0.05$, #, $p<0.0001$ Vehicle - Vehicle vs Vehicle-MK-801, §, Vehicle – MK-801 vs MK-801 – NAN-190, Sidak’s post-hoc). Time bins shown represent 5-minute intervals.

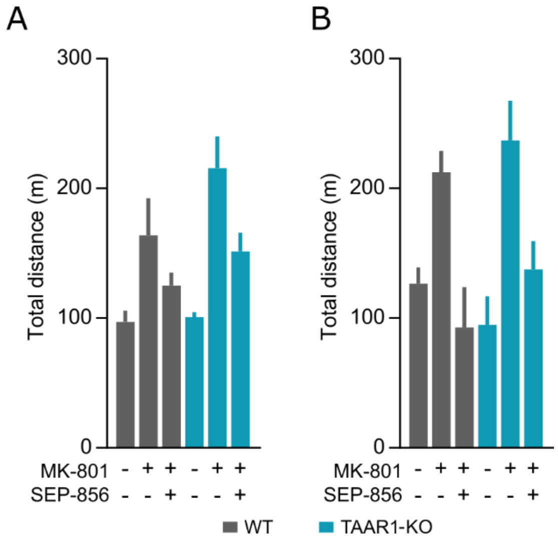


**Supplementary Figure 8: Lack of sex differences in responsiveness to MK-801 induced hyperlocomotion**

Mice from Figure 5D were separated into male (A, n=4) and female (B, n=3) WT and TAAR1-KO mice.

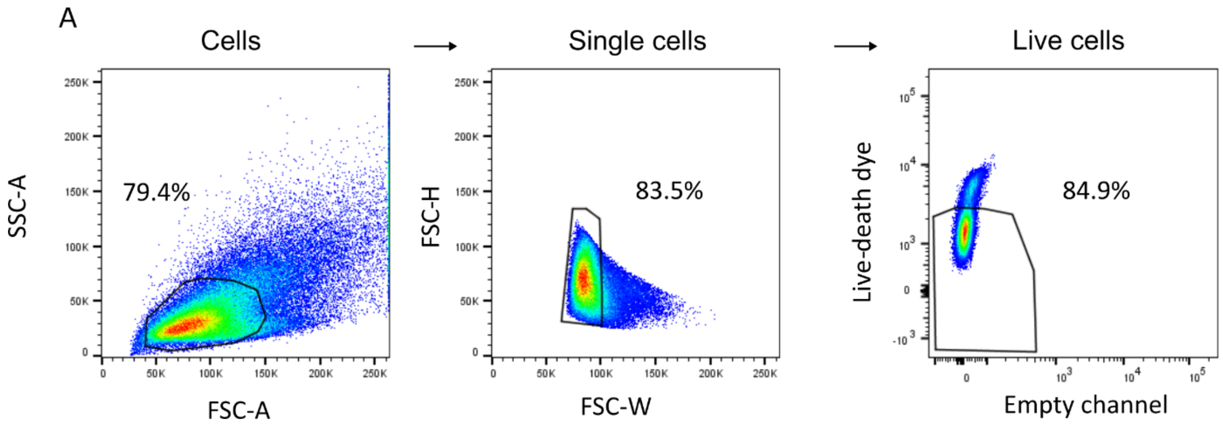


**Supplementary Figure 9: Gating strategy for flow cytometry**

(A) Single cells were sorted and selected using a live/death dye. Only living cells at the time of fixation were considered for subsequent quantifications of HA antibody staining.

**Supplementary References:**

1. Di Cara B, Maggio R, Aloisi G, Rivet J-M, Lundius EG, Yoshitake T, et al. Genetic Deletion of Trace Amine 1 Receptors Reveals Their Role in Auto-Inhibiting the Actions of Ecstasy (MDMA). J Neurosci. 2011;31:16928 LP – 16940.
2. Jespersen T, Grunnet M, Angelo K, Klaerke DA, Olesen SP. Dual-function vector for protein expression in both mammalian cells and Xenopus laevis oocytes. vol. 32. England; 2002.
3. Wan Q, Okashah N, Inoue A, Nehmé R, Carpenter B, Tate CG, et al. Mini G protein probes for active G protein–coupled receptors (GPCRs) in live cells. J Biol Chem. 2018;293:7466–7473.
4. Nehmea R, Carpenter B, Singhal A, Strege A, Edwards PC, White CF, et al. Mini-G proteins: Novel tools for studying GPCRs in their active conformation. PLoS One. 2017;12:1–26.
5. Sahlholm K, Barchad-Avitzur O, Marcellino D, Gómez-Soler M, Fuxe K, Ciruela F, et al. Agonist-specific voltage sensitivity at the dopamine D2S receptor--molecular determinants and relevance to therapeutic ligands. Neuropharmacology. 2011;61:937–949.
6. Ågren R, Sahlholm K. Voltage-Dependent Dopamine Potency at D(1)-Like Dopamine Receptors. Front Pharmacol. 2020;11:581151.
